# Skeletal and dentoalveolar effects on the midpalatal suture and maxillary arch assessed by occlusal radiographs and three-dimensional digital models in patients treated with invisalign palatal expander and rapid palatal expander: a pilot study

**DOI:** 10.3389/fdmed.2026.1757094

**Published:** 2026-03-18

**Authors:** Luca Levrini, Stefano Saran, Emanuela Imbesi, Irene Vanini, Veronica Russo, Valeria Rimoldi, Andrea Carganico, Nicola Giannotta, Martina Perugini

**Affiliations:** 1 Country Department of Human Sciences, Innovation and Territory, Post Graduate School in Orthodontics, University of Insubria, Varese, Italy; 2 Department of Human Sciences, Innovation and Territory, School of Dentistry, University of Insubria, Varese, Italy; 3 School of Dental Medicine, University of Insubria, Varese, Italy

**Keywords:** invisalign palatal expander, invisalign, IPE, palatal expansion, rapid palatal expander

## Abstract

**Introduction:**

Maxillary transverse deficiency (MTD) is a common craniofacial condition associated with posterior crossbite, dental crowding, and compromised respiratory function. This study aimed to evaluate whether the Invisalign Palatal Expander (IPE) can induce midpalatal suture opening and occlusal changes, and to compare these outcomes with those obtained using conventional rapid palatal expansion (RPE).

**Materials and methods:**

Thirty subjects (14 females, 16 males; aged 6–18 years) with mixed dentition were enrolled and divided into two groups: 15 treated with IPE and 15 with RPE. Radiographic images and digital dental models were analyzed before (T0) and after treatment (T1) to assess skeletal and occlusal changes, including midpalatal suture opening and transverse arch dimensions. Molar tipping and palatal depth were also measured. Patient-reported side effects during the first month of treatment were evaluated using a questionnaire addressing bulkiness, tongue impression, dysphonia, dysphagia, and gag reflex, scored on a 1–5 scale. Inclusion criteria comprised mixed dentition, erupted first molars, deciduous fourth/fifth teeth or erupting premolars, and complete diagnostic records. Exclusion criteria included previous orthodontic treatment, craniofacial anomalies, extraction therapy, allergies, hereditary angioedema, or active caries.

**Results:**

No significant differences were found between groups in the number of activations, radiographic outcomes, or theoretical expansion, indicating comparable skeletal effects. Midpalatal suture opening was achieved in all patients. Baseline occlusal conditions were similar between groups. After normalization for the number of activations, statistically significant differences were observed for arch depth, canine gingival width, canine dental width, and arch perimeter, all of which were greater in the RPE group (*p* < 0.05). No significant differences were found in intermolar angle or palatal depth changes. Questionnaire analysis revealed no significant differences in reported side effects between groups.

**Conclusions:**

The Invisalign Palatal Expander effectively produced transverse maxillary expansion with midpalatal suture opening, yielding skeletal and occlusal outcomes comparable to those of the Hyrax expander. Although the amount of expansion was slightly lower, the IPE demonstrated more controlled and predictable results, supporting its use as a valid alternative in mixed dentition and as a step toward fully digital orthodontic protocols.

## Introduction

Maxillary transverse deficiency (MTD) is one of the most prevalent skeletal discrepancies in the craniofacial region, with a prevalence of 21% in patients during mixed dentition (1–3). This condition represents a frequent orthodontic malocclusion that can manifest at any age and may be skeletal, dental, or a combination of both (4, 5). Due to its early onset, MTD can interfere with proper craniofacial development, potentially resulting in functional and aesthetic consequences if left untreated (6).

Posterior crossbite is one of the main clinical expressions of MTD, affecting 8%–23% of children in primary and mixed dentitions (5, 7–12), with prevalence varying across populations (13). Clinically, it leads to occlusion of the upper vestibular cusps into the fossae of the lower posterior teeth, producing a functional shift during mastication (14–17). Other manifestations include dental crowding, dental protrusion, buccal corridors, high palatal vault, elevation of the nasal floor, and increased nasal airway resistance (1, 18–22).

The etiology of malocclusion is complex and multifactorial, involving both genetic and environmental factors, including thumb sucking, mouth breathing, low tongue posture, and prolonged pacifier use (19). Early diagnosis and intervention are critical to prevent long-term craniofacial complications and to improve overall oral and respiratory health (10, 19).

MTD is typically managed through palatal expansion, which aims to widen the narrow maxillary arch by separating the midpalatal suture (4, 13). Palatal expansion techniques are usually categorized into rapid maxillary expansion (RME) and slow maxillary expansion (SME), based on the magnitude of force and the interval of screw activation. RME involves applying high forces (about 100 N) over a short period, with the expansion screw typically activated once or twice daily, resulting in 0.25–0.5 mm of expansion per day. Conversely, SME uses lighter, continuous forces (around 20 N), with screw activation once or twice per week, achieving 0.25 mm of expansion per week, allowing for more gradual skeletal adaptation and potentially more stable results (3, 5, 15, 18, 23).

Clinically, the appliances used for these techniques are broadly classified as fixed or removable. Fixed devices, such as Haas and Hyrax expanders, can be employed for both RME and SME, whereas removable plates are generally used for SME, and their effectiveness largely depends on patient compliance (4, 7, 8, 14). Removable appliances are a valid therapeutic alternative to fixed devices when patient cooperation is adequate. They can produce skeletal effects, provide better palatal support, and cause less damage to the teeth. Patients have also reported lower levels of discomfort during the initial phase of treatment (4, 5). In recent decades, there has been a growing demand for more comfortable orthodontic treatment options as alternatives to traditional fixed appliances (24, 25).

A novel device, the IPE, has recently been introduced by Align Technology. The IPE consists of a series of 3D-printed expanders made of nylon polyamide 12, anatomically designed to conform to the palatal curvature and cover the clinical crowns of the upper first deciduous molar (or first premolar), second upper deciduous molar (or second premolar), and first upper permanent molar. IPE treatment involves an active expansion phase lasting approximately 30 days, with each unit producing an activation of 0.25 mm. The treatment protocol also includes a retention phase using a device called the “Palatal Holder,” designed to maintain the expansion achieved during the active phase. The Palatal Holder is worn for 12 weeks (23, 26–28).

According to Align Technology Inc., the IPE is capable of producing skeletal expansion of the maxilla. However, to date, no studies have evaluated its efficacy in achieving midpalatal suture separation.

The aim of this study is to evaluate the effects of IPE treatment, with particular focus on determining whether the device induces midpalatal suture opening, whether occlusal changes occur and, if so, characterizing the specific variations, and assessing the presence of any side effects or complications. These outcomes were compared with those observed in patients treated with a rapid palatal expander (RPE) to provide a comprehensive understanding of the clinical implications of each intervention.

## Materials and methods

The parents of the patients signed an informed consent form, and no financial incentive was given for participation. The research was approved by the ethics committee n. 0111335 of “Università degli Studi dell'Insubria”, Varese, Como, Italy.

### Sample

A total of 33 patients (16 females and 17 male) were prospectively recruited at the dental department of the “Ospedale di Circolo, fondazione Macchi”, University of Insubria, Varese, Italy. Patients were divided into two groups: 17 cases treated with IPE and 16 controls treated with RPE. Inclusion and exclusion criteria were defined according to the current evidence in the literature (3).

The inclusion criteria for this study were:

- Age: between 7 and 18 years
- Mixed dentition
- Presence of erupted maxillary first molars and deciduous first and second molars, or erupting premolars, with at least 4 mm of clinical crown available for appliance anchorage
- Clinical indication for maxillary arch expansion

The exclusion criteria for this study were:

- Previous orthodontic treatment
- Presence of craniofacial anomalies or syndromes (e.g., cleft lip and palate, dental agenesis affecting anchorage teeth)
- Cases requiring dental extractions
- Allergy to polyamide-12
- Hereditary angioedema, this criterion was adopted in accordance with the manufacturer's safety guidelines for the Invisalign® Palatal Expander System. HAE may predispose to rapid swelling, including of the larynx, and thus represents a potential risk when using the device (27).
- Active carious lesions involving anchorage teeth

### Exclusions and limitations in measurements

Complete and adequate diagnostic records were required for data analysis, including digital dental models and radiographic records acquired at both T0 and T1. These methodological requirements were predefined in the study protocol to ensure a reliable assessment of transverse, occlusal, and skeletal changes.

During the evaluation at T1, two patients from the experimental group (one male and one female) were excluded because the required T1 radiographic records were missing, which prevented accurate assessment of the study outcomes. In addition, one female patient from the control group was excluded because exfoliation of tooth 5.5 during treatment compromised appliance anchorage and resulted in non-compliance with the predefined clinical inclusion criteria.

Therefore, the final sample analyzed in this study consisted of 30 patients (14 females and 16 males): 15 treated with the IPE and 15 treated with RPE.

### Devices description

IPE consists of a series of 3D-printed polyamide-12 (Nylon 12) expanders that follow the curvature of the palate, contacting the lateral palatal walls, but not the roof of the palate and cover the three posterior teeth: the first deciduous molar (or first premolar), the second deciduous molar (or second premolar), and the first permanent molars.

At the buccal level, the expanders are 0.75 mm thick, increasing to 1.5 mm at the occlusal surfaces. In the palatal region, the thickness ranges from 2.5 mm up to 3 mm.

Approximately one month of active expansion is performed, and the number of devices required varies depending on the amount of expansion desired and the severity of the case. A new aligner is changed every 24–48 h, and in non-compliant patients, every 36 h, and each one has an activation of 0.25 mm. The device is used all day, including during meals, and removed only during oral hygiene (IOD) and to clean the device. The first expander is passive to help the patient get used to wearing it.

The treatment includes a retainer, the Palatal Holder, which is used to maintain the expansion achieved in the upper jaw. It is used for 12 weeks (3 months) and replaced every 2–4 weeks. The retainer is removed only during meals (26).

In this study the Hyrax appliance was adopted in the control group. The Hyrax appliance is a fixed orthodontic device designed to achieve rapid maxillary expansion (RME) by applying lateral forces to the maxillary bones. It is one of the most widely used palatal expanders in orthodontics and is particularly effective in growing patients for correcting transverse maxillary deficiencies. The Hyrax appliance consists of a central expansion jackscrew that serves as the force-generating unit. The central screw is connected to four rigid support arms, two of which is welded to band ring. For the Hyrax appliance, one quarter-turn of the central screw (approximately 0.225 mm per activation) is performed once per day. The total number of activations depends on the severity of the maxillary constriction and continues until the desired expansion is achieved (29–31).

### Study design

In this prospective study both groups were evaluated radiographically with occlusal x-rays and digital models.

Occlusal x-rays were taken at T0, before the start of treatment, and at T1, at the end of active expansion. The x-ray was performed by adjusting the headrest to make the maxillary arch parallel to the floor. The vertical midline of the face should be perpendicular to the floor.

The occlusal film (5.7 × 7.6 cm) is centred in the patient's mouth, directing it on the arch. The long axis of the film is positioned longitudinally, perpendicular to the midline of the arch, and one side of the film is placed against the upper teeth. Next, it is asked the patient to gently close against the film to hold it in place. The upper edge of the cone was positioned between the eyebrows with a vertical angulation of 65 degrees. The correct horizontal angulation is achieved by setting the central beam parallel to and through the midline of the arch until it reaches the center of the film (32).

Since the distance between the x-ray tube and the film may vary, a proportional measurement method was used. Specifically, the length of the incisal margin of tooth 2.1 was measured directly on the patient and then compared to the same measurement on the radiographic film using a digital ruler. This allowed to calculate the proportion between the actual size and the radiographic size, in order to correct for possible distortions caused by variations in the radiographic set-up.

**Figure 1:**
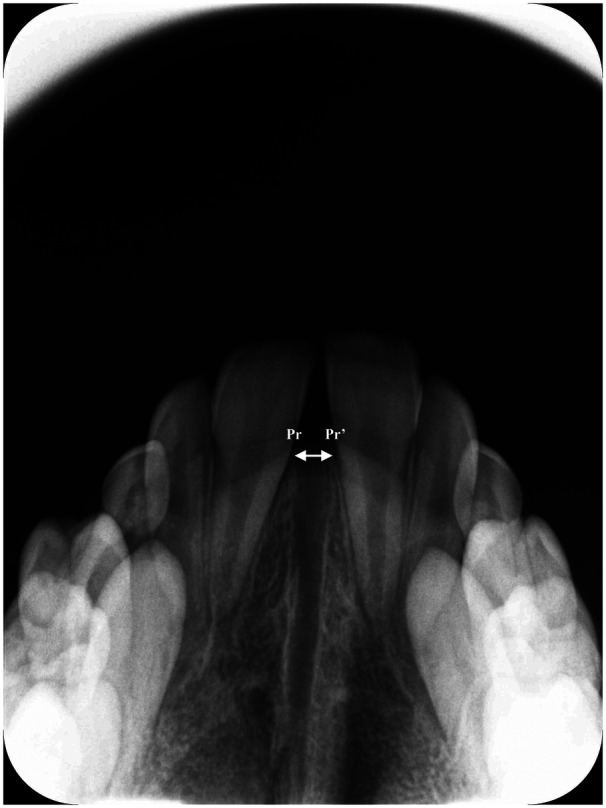
The amount of midpalatal suture separation was quantified by calculating the distance between the prosthion points (Pr–Pr′) on maxillary occlusal radiographs.

**Figure 2:**
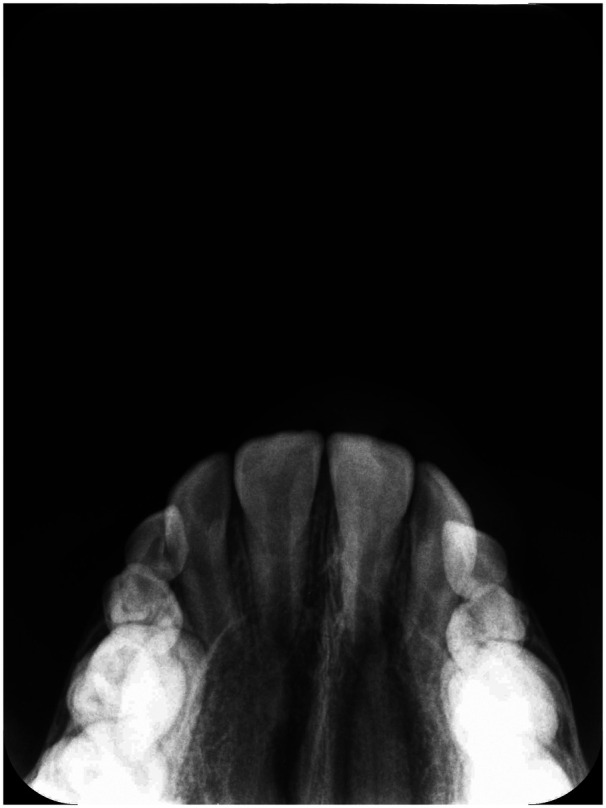
Occlusal radiograph at T0 in a patient treated with IPE.

**Figure 3:**
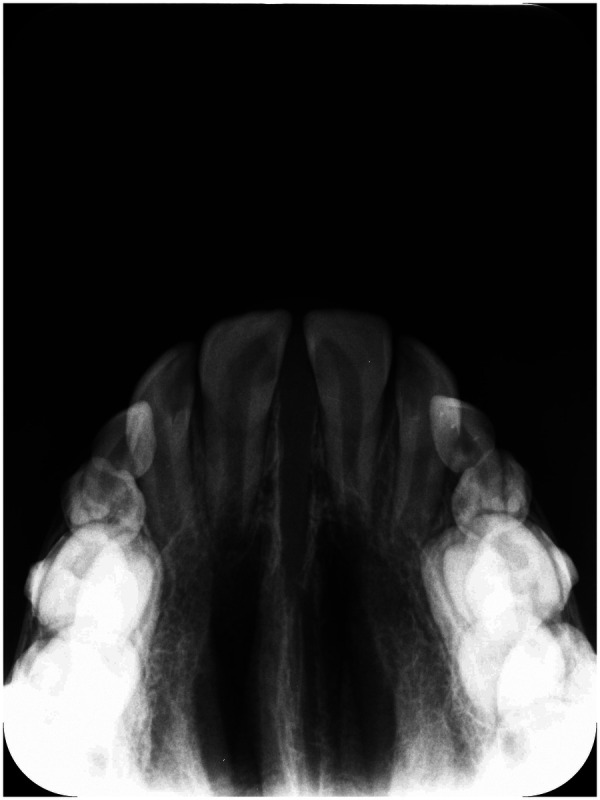
Occlusal radiograph at T1 in a patient treated with IPE.

To evaluate transverse skeletal changes, the width of the midpalatal suture opening was directly assessed by measuring the linear distance between the prosthion landmarks (Pr–Pr′) on maxillary occlusal radiographs acquired before and after the conclusion of the active expansion phase (33, 34) (Figures 1–3). This analysis allowed radiographic confirmation of the skeletal effects achieved through the expansion protocol.

To standardize the terminology, the term “activation” was subsequently adopted to indicate either one-quarter of a Hyrax screw turn or a single IPE appliance. It should be noted that each IPE appliance and each Hyrax screw quarter-turn correspond to a transverse increment of 0.25 mm; the actual value of expansion per activation of Hyrax is 0.225 mm, but was rounded for simplicity in calculations.

For each occlusal radiograph taken before (T0) and after treatment (T1), the following parameters were measured:

Margin: radiographic measurement of the margin of tooth 2.1 at T0 and T1.
Suture: radiographic distance between the reference points Pr and Pr′, representing the opening of the palatal suture, measured at T0 and T1.
Real margin: measurement of the margin of tooth 2.1 obtained from the digital model (STL), considered the most reliable anatomical reference.
Real suture: real opening of the suture calculated at T0 and T1, obtained using a proportion that applies the real margin as a correction factor for the radiographic measurements.
Expansion ratio: ratio between the real suture opening and the predicted theoretical expansion (number of Hyrax screw turns or number of IPE devices used).
*Δ* Rx/activations: radiographic change of the suture between T1 and T0, divided by the number of Hyrax screw turns or IPE devices used.
*Δ* Suture/activations: change in the real suture between T1 and T0 (after proportional correction), divided by the number of turns or devices used in the two groups.

**Figure 4:**
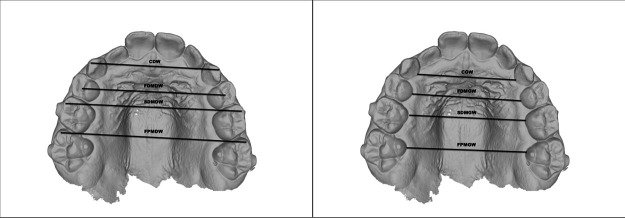
Arch width linear measurements. **(A)** Cuspids level: canine gingival width (CGW), first deciduous molar gingival width (FDGW), second deciduous molar gingival width (SDGW), first permanent molar gingival width (FPMGW). **(B)** Gingival level: canine dental width (CDW), first deciduous molar dental width (FDMDW), second deciduous molar dental width (SDMDW), first permanent molar dental width (FPMDW).

**Figure 5:**
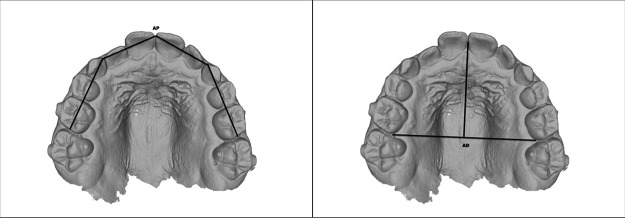
**(A)** Arch perimeter, **(B)** arch depth.

**Figure 6:**
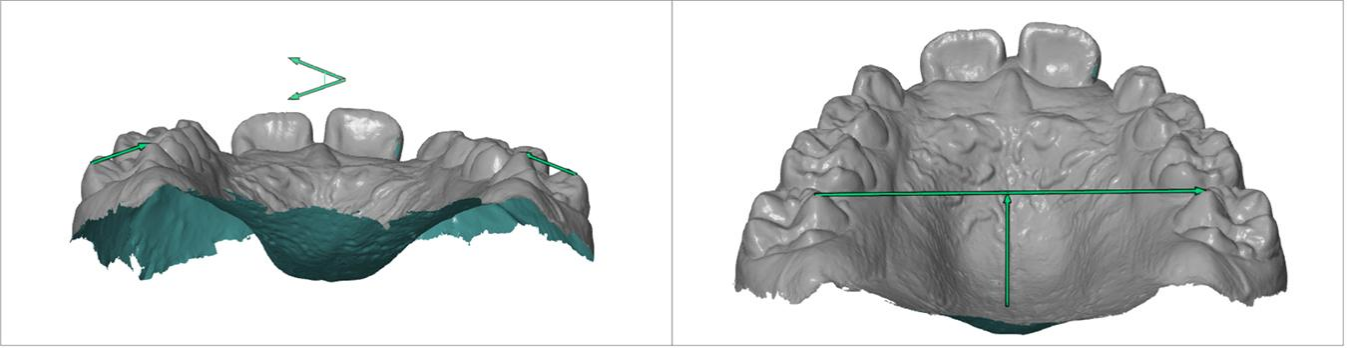
**(A)** Intermolar angle, **(B)** palatal depth.

Whereas, for the evaluation of the occlusal component (Figures 4–6), the following measurements were recorded on digital models created by the Geomagic Design 2016 software, based on iTero intra- oral scanner:

Canine gingival width (CGW): linear distance between the center of the palatal surface of the upper canines in contact with the gingival margin.
First deciduous molar gingival width (FDGW): linear distance between the center of the palatal surface of the first deciduous upper molars in contact with the gingival margin.
Second deciduous molar gingival width (SDGW): linear distance between the inner point of the palatal surface of the first deciduous upper molars in contact with the gingival margin.
First permanent molar gingival width (FPMGW): linear distance between the inner point of the palatal surface of the first permanent upper molars in contact with the gingival margin.
Canine dental width (CDW): linear distance between the cusp tips of the upper deciduous canines.
First deciduous molar dental width (FDMDW): linear distance between the cusp tips of the deciduous upper first molars.
Second deciduous molar dental width (SDMDW): linear distance between the mesio-buccal cuspids of the second deciduous upper molars.
First permanent molar dental width (FPMDW): linear distance between the mesio-buccal cuspids of the first permanent upper molars.
Arch perimeter (AP): length of a line passing through the mesial aspect of first permanent upper molars, mesial aspect of deciduous upper canines and mesial aspect of central incisors.
Arch depth (AD): length of a perpendicular line constructed from the mesial contact points of the central incisors to a line connecting the mesial contact points of the first molars (2, 3, 35).

In addition to the main parameters, two further variables were measured: molar tipping and palatal depth. The measurements were performed using the Zeiss Inspect Suite 2025 3D metrology software, onto which the pre-treatment (T0) and post-treatment (T1) STL files of digital scans were uploaded.

Intermolar Angle (IMA): Molar tipping was measured selecting as reference points the distobuccal and mesiopalatal cusps of teeth 1.6 and 2.6. Tangent lines were drawn through these points, and the angle formed at their intersection defined the molar inclination. The tipping variation was obtained by subtracting the angle measured on the T0 STL model from that measured on the T1 STL model, thus representing the change in molar tipping following treatment.

Palatal depth: Palatal depth was measured by drawing a line connecting the mesiopalatal cusps of teeth 1.6 and 2.6, which was then intersected by a perpendicular line passing through the midsagittal point of the palate. The linear distance (mm) between these two lines represented the palatal depth. The variation in this parameter was calculated by subtracting the pre-treatment (T0) measurement from the post-treatment (T1) value, thereby quantifying the treatment-related change in palatal depth (36).

Measurements were taken twice, by different operator, on occlusal x-ray and on the STL file.

All patients were administered a questionnaire assessing the side effects associated with using the device (for both ERP and IPE) during the first month of treatment. Five questions were specifically asked: The first concerned the size of the device in the patient's oral cavity. The second question asked whether the device has left an imprint on the tongue. The third question concerned dysphonia, whether the patient has difficulty to speaking or pronouncing letters. The fourth question concerned dysphagia, difficulty swallowing and ingesting certain foods and the final question concerned the gag reflex, whether the patient has a gag reflex with the device applied to the palate. Patients respond to these questions by assigning a score from 1 to 5 based on the severity of discomfort, where 1 corresponds to no discomfort, 2 is mild, 3 is moderate, 4 is severe and 5 is extreme.

## Statistical analysis

In this study, the measured values were taken into consideration, and the normality of the data was evaluated using the Shapiro–Wilk test. For normally distributed variables, a parametric test was applied: a *T* test for independent samples to compare groups. For variables that did not follow a normal distribution, the Mann–Whitney *U* test was employed.

To assess inter-observer reliability, 20% of the sample was independently re-measured by a second examiner after a 7-day interval. The intraclass correlation coefficient (ICC) was used to evaluate measurement reliability.

## Sample size

The sample of the present study comprised fifteen patients per group. Given the exploratory nature and novelty of the investigation, the study can be considered a pilot. Larger-scale studies will be necessary to confirm and validate the findings. Nevertheless, a sample size calculation was performed based on the study by Bistaffa AGI et al. (37), which compared the magnitude of suture opening among different devices. Assuming a mean suture opening of 2.22 (SD = 0.48) for the Haas device and 2.58 (SD = 0.55) for the Hyrax device, a total sample size of 56 patients would be required to achieve 80% statistical power with an alpha level of 0.05 (38).

## Results

A total of 33 patients (16 females, 17 males) aged 7–18 years were initially enrolled. Three patients were excluded at T1: two due to lack of radiographic material and one due to an early loss of a dental element, resulting in a final sample of 30 subjects (14 females, 16 males). Participants were divided into two groups of 15: the Hyrax group (10 females, 5 males) and the IPE group (4 females, 11 males).

Data distribution was assessed using the Shapiro–Wilk test. Variables with a normal distribution were analyzed using independent samples *t*-tests, whereas non-normally distributed variables were compared using the Mann–Whitney *U* test. Results are reported as mean ± standard deviation. Detailed descriptive statistics and additional data are reported in Supplementary Tables S1–S6.

The mean age of participants at baseline was 8.27 ± 1.33 years in the Hyrax group and 9.53 ± 2.80 years in the IPE group, with no statistically significant difference (Mann–Whitney *U* test, *p* > 0.05) (Table 1).

**Table 1:** Baseline age distribution of the two study groups.

Group	*N*	Mean ± DS	Median	Min-Max	95% CI	Shapiro–Wilk
Hyrax	15	8.62 ± 1.33	8.8	8–11	8.24–9.36	W = 0.729 (*p* = 0.0005)
IPE	15	9.53 ± 2.80	9.8	7–18	8.41–11.19	W = 0.610 (*p* = 0.0002)

The mean number of activations was 23.87 ± 5.74 in the Hyrax group and 21.20 ± 3.86 in the IPE group, with no significant intergroup difference (*p* > 0.05) (Table 2).

**Table 2:** Distribution of activations in patients treated with Hyrax or with IPE.

Variables	*N*	Mean ± DS	Median	Min-Max	95% CI	Shapiro–Wilk
N° Hyrax screw turns	15	23.87 ± 5.74	22.0	15–35	20.69–27.0470	W = 0.95(*p* = 0.48)
N° IPE devices	15	21.20 ± 3.86	20.00	17–30	19.06–23.33	W = 0.83(*p* = 0.01)

## Radiographic analysis

**Figure 7:**
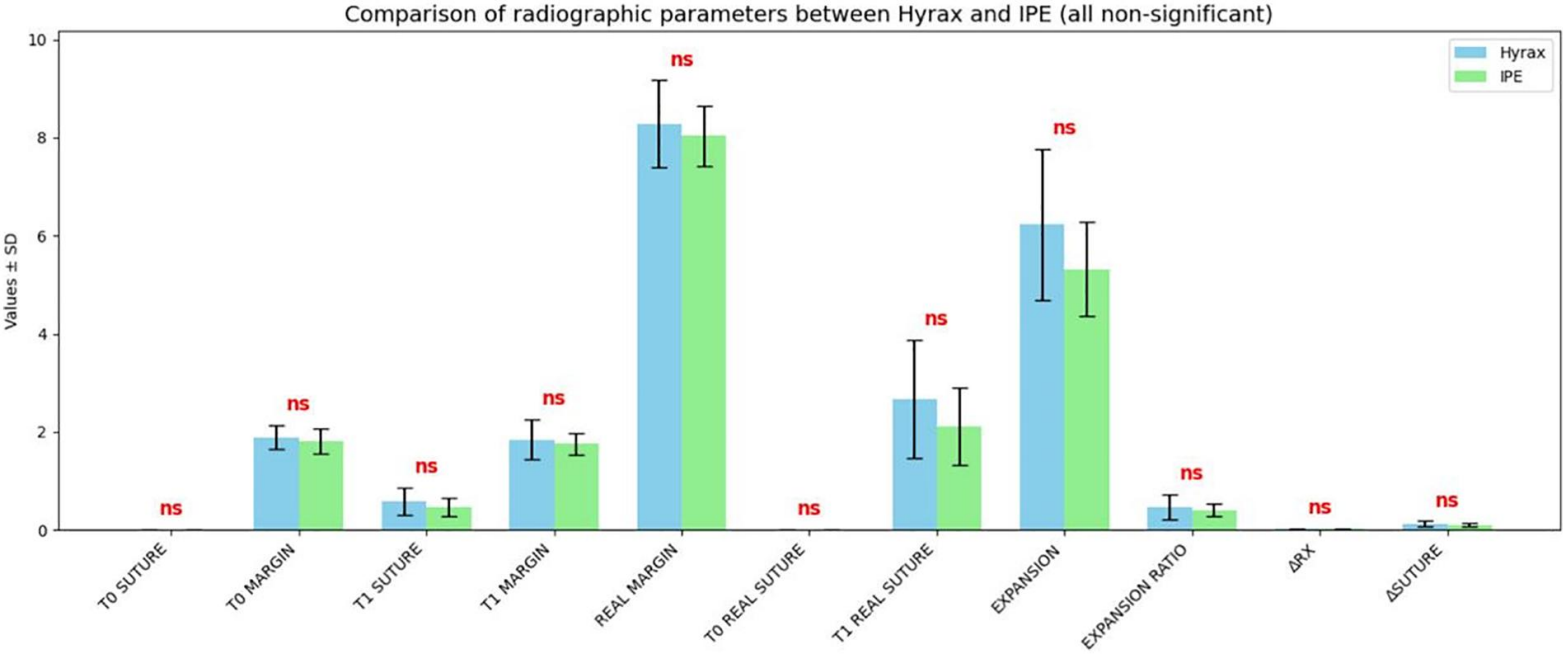
HISTOGRAM: comparison between the mean values of the radiographic parameters measured with the IPE and Hyrax expanders. The blue bars represent the values obtained with the Hyrax expander, whereas the green bars indicate the values measured with the IPE expander.

Radiographic parameters were generally normally distributed; non-normal distributions and detailed results are reported in Supplementary Tables S1, S2. No statistically significant differences were observed between groups for any parameter. The largest mean difference was 0.54 mm for T1 Real Suture, which was not significant (*p* = 0.178) (Figure 7).

## Occlusal analysis

Baseline occlusal parameters were comparable between groups, confirming that the groups were initially homogeneous (Supplementary Tables S3, S4). Δ values (T1–T0) were calculated for each parameter and normalized by the number of activations in each group to account for differences in treatment units. Normalization was calculated using the following formula: normalized value = *Δ* parameter/tratment unit. In this way, the *Δ* values of the occlusal parameters were standardized—that is, expressed in the same unit of measurement or relative to a common reference—allowing the differences observed between the groups to be mainly attributed to the treatment effect rather than external factors. Normalized Δ values are reported in Tables 3, 4.

**Table 3:** Delta occlusal parameters normalized by the number of activations.

Hyrax Group					
Parameter	*N*	Min	Max	Mean	SD
CGW	15	0.06	0.40	0.21	0.10
FDMGW	15	0.08	0.40	0.23	0.09
SDMGW	15	0.05	0.42	0.21	0.11
FPMGW	15	0.01	0.39	0.16	0.11
CDW	15	0.03	0.41	0.21	0.10
FDMDW	15	0.09	0.58	0.24	0.13
SDMDW	15	0.12	0.44	0.25	0.11
FPMDW	15	0.08	0.40	0.20	0.09
AP	15	0.03	0.44	0.22	0.12
AD	15	−0.35	0.03	−0.11	0.12

**Table 4:** Delta occlusal parameters normalized by the number of devices.

IPE Group					
Parameter	*N*	Min	Max	Mean	SD
CGW	15	0.04	0.18	0.11	0.04
FDMGW	15	0.08	0.26	0.19	0.05
SDMGW	15	0.02	0.27	0.19	0.06
FPMGW	15	0.08	0.26	0.19	0.05
CDW	15	0.02	0.29	0.13	0.06
FDMDW	15	0.09	0.29	0.21	0.06
SDMDW	15	0.09	0.48	0.24	0.09
FPMDW	15	0.12	0.30	0.22	0.05
AP	15	0.02	0.21	0.11	0.06
AD	15	−0.14	0.09	−0.03	0.07

**Figure 8:**
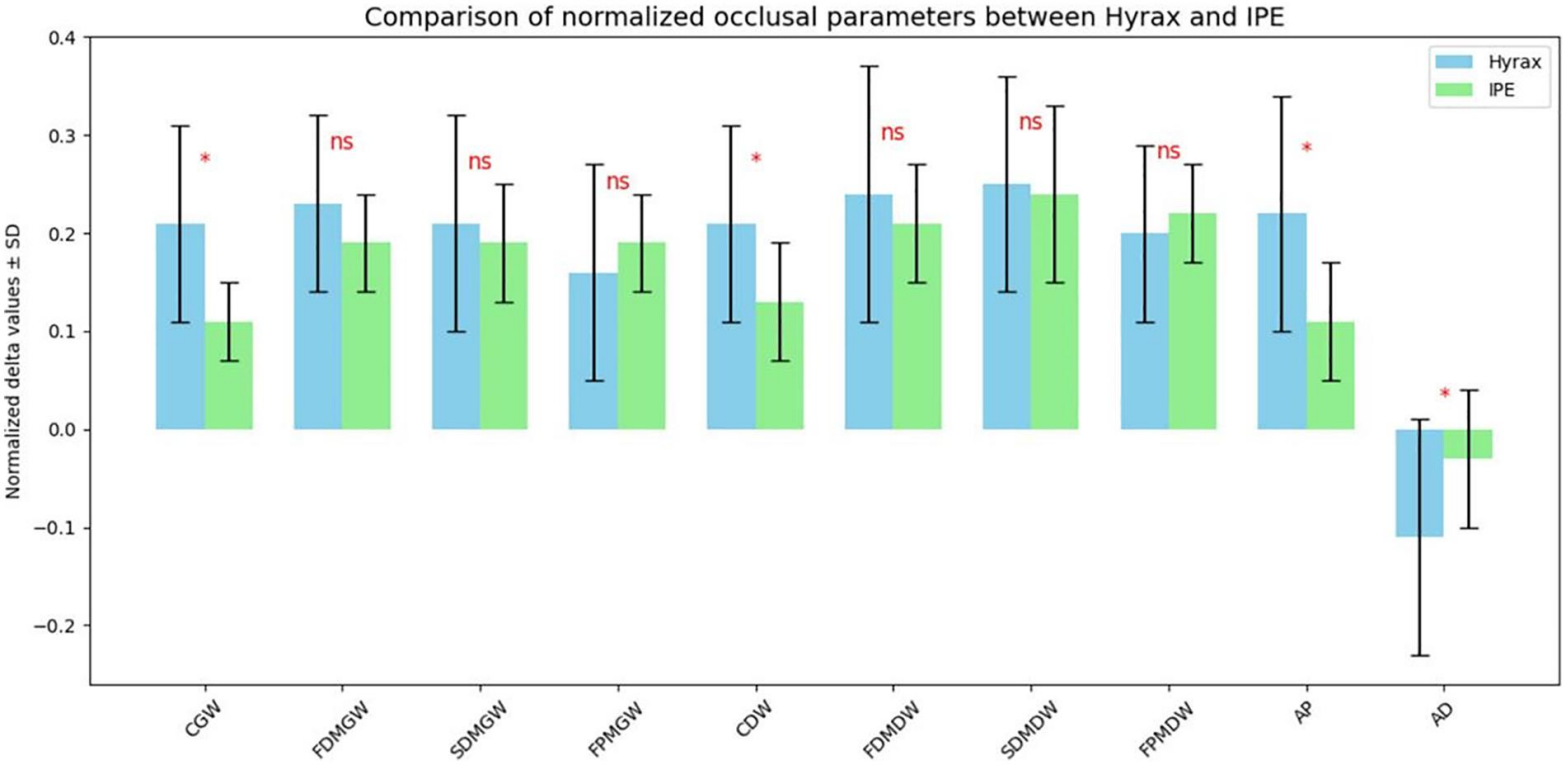
HISTOGRAM: comparison between the normalized mean A values of the occlusal parameters in the IPE and Hyrax groups. The blue bars represent the values obtained with the Hyrax expander, whereas the green bars indicate the values measured with the IPE expander.

Among non-normally distributed parameters, only AD showed a statistically significant difference between groups (*p* = 0.036), whereas SDMGW and SDMDW did not differ significantly. For normally distributed parameters, the Hyrax group exhibited significantly higher CGW, CDW, and AP compared to the IPE group (Figure 8).

In a subsequent phase of the analysis, the variables Intermolar Angle (IMA) and Palatal Depth (PD) were included, measured in both groups at T0 and T1. For each variable, the variation value (*Δ* = T1−T0) was also calculated to quantify the change over time (Tables 5, 6).

**Table 5:** Occlusal parameters in Hyrax group.

Hyrax Group					
Parameter	*N*	Min	Max	Mean	SD
T0 intermolar angle	15	16.14	44.43	32.18	8.45
T1 intermolar angle	15	19.26	47.15	3,685	7.40
Δ intermolar angle	15	0.30	8.01	4.67	2.21
T0 palatal depth	15	14.374	19.74	16.09	1.42
T1 palatal depth	15	13.60	18.14	15.26	1.32
Δ palatal depth	15	−1.99	−0.082	−0.82	0.63

**Table 6:** Occlusal parameters in IPE group.

IPE Group					
Parameter	*N*	Min	Max	Mean	SD
T0 intermolar angle	15	18.55	46.520	32.70	9.10
T1 intermolar angle	15	21.60	53.38	39.29	9.34
*Δ* intermolar angle	15	2.17	12.67	6.59	3.25
T0 palatal depth	15	14.104	20.33	16.023	1.73
T1 palatal depth	15	12.45	19.76	15.36	1.80
Δ palatal depth	15	−2.18	−0.147	−0.66	0.57

Comparisons between the IPE and ERP groups showed no statistically significant differences for any of these variables. Specifically, IMA at T0 and T1, as well as *Δ*IMA, were analyzed using independent samples *t*-tests and showed *p*-values of 0.872, 0.433, and 0.070, respectively. Regarding palatal depth (PD), the Shapiro–Wilk test indicated a normal distribution for PD at T1, while PD at T0 and *Δ*PD (T1–T0) were non-normally distributed. Accordingly, PD at T1 was compared between groups using an independent samples *t*-test, showing no significant difference (*p* > 0.05). PD at T0 and *Δ*PD were analyzed with the Mann–Whitney *U* test, which also revealed no significant differences between groups (*p* = 0.65 and *p* = 0.512, respectively).

The analysis demonstrated excellent inter-observer agreement for all assessed variables, with ICC values exceeding 0.97.

## Questionnaire analysis

Questionnaire variables, including Bulkiness, Tongue Impression, Dysphonia, Dysphagia, and Gag Reflex, are reported in Supplementary Tables S5, S6. All variables showed non-normal distributions (Shapiro–Wilk test), and group comparisons were performed using the Mann–Whitney *U* test. No statistically significant differences were observed between the Hyrax and IPE groups for any of the variables. Although the Hyrax group showed slightly higher mean values for Dysphagia (2.73 vs. 2.50) and Dysphonia (2.33 vs. 1.93), these differences were not statistically significant.

## Discussion

The present study aimed to compare the skeletal, dental and clinical effects of the IPE with those of the conventional Hyrax appliance in a sample of growing patients.

The final sample included 30 subjects (14 females and 16 males). At baseline (T0), the two groups had comparable characteristics, therefore the differences observed at the end of treatment can be attributed primarily to the effects of the appliances used.

The results of this pilot study suggest that both the Hyrax expander and the IPE were effective in producing transverse widening of the maxillary arch, with no statistically significant differences for most of the occlusal and radiographic parameters evaluated.

Hyrax is a rapid maxillary expander which, by delivering high forces, causes significant skeletal expansion, but also unwanted dentoalveolar movements. In contrast, IPE represents an innovative system that acts in a more localized way with more controlled forces.

In the present study, the skeletal, dentoalveolar and clinical effects were evaluated using diagnostic tools validated in the literature. The opening of the midpalatal suture was analyzed via maxillary occlusal radiography, a method already validated by Ciambotti et al. (3, 13), while the occlusal and dentoalveolar measurements were performed on digital models using the Geomagic Design 2016 software, previously validated on physical and digital models (3, 39). The evaluation was also complemented by direct and indirect evaluation tools.

Midpalatal suture opening was observed in both experimental groups. In the IPE group, the mean real suture expansion at T1 (2.12 ± 0.79 mm) was comparable to that achieved with the Hyrax expander (2.67 ± 1.20 mm), with no statistically significant difference (*p* = 0.178). These results are consistent with findings by Alves et al., who demonstrated that alternative expansion appliances can also induce orthopedic effects, particularly in the anterior suture region. Moreover, the narrower 95% confidence interval observed for the IPE group (1.69–2.56 mm) compared with Hyrax (2.00–3.33 mm) suggests greater predictability and clinical consistency of expansion with the IPE, likely due to its more controlled and gradual activation pattern (8, 34, 39)..

An important clinical aspect concerns the ΔRx/activations and Δsuture/activations: in the IPE group, the mean values were 0.02 ± 0.01 mm and 0.10 ± 0.03 mm, respectively, while in the Hyrax group mean values were 0.02 ± 0.01 mm and 0.12 ± 0.06 mm. The close similarity of these parameters confirms that the IPE can transmit adequate forces to the midpalatal suture, inducing clinically relevant skeletal expansion. It is important to note that not all expansion protocols produce a radiographically visible separation of the midpalatine suture, as highlighted in the studies by Hansson et al. and Sandıkçıoğlu where both the Quad Helix and Expansion plates either do not induce suture opening or induce it only partially (8, 11).

This study shows that the IPE seems to be capable of producing occlusal and dentoalveolar changes comparable to those achieved with the Hyrax. In the posterior region the analysis of intermolar widths (FDMDW, SDMDW, and FPMDW) revealed nearly overlapping values between the two groups, in fact no statistically significant differences were found for these parameters (*p* > 0.05). This finding too is consistent with the literature, which shows that slow or removable expansion devices can achieve significant molar-level increases (2, 10, 13).

While in the anterior region, at the canine level, the effect of Hyrax was superior, showing statistically significant differences for both the CGW and CDW parameters. In the Hyrax group, the CGW parameter exhibited a mean normalized increase of 0.21 ± 0.10 and 0.11 ± 0.04 in the IPE group. Similarly, the CDW increased by 0.21 ± 0.10 in the Hyrax group compared to 0.13 ± 0.06 in the IPE group.

The observed difference in the anterior region can be explained by the specific design of the IPE, which engages only the three posterior elements: the permanent first molar, E (or second premolar), and D (or first premolar), leaving the canine region unaddressed. Consequently, force transmission is not uniform across the entire arch, and expansion tends to be concentrated in the posterior region.

Comparison with the literature supports this interpretation. In particular, Bruni et al. reported that the inter-canine increase achieved with RME does not differ significantly from that obtained with aligners (CAT), although it is on average slightly greater. This finding aligns with the preliminary results of the study, where the Hyrax produced greater anterior expansion, while the IPE resulted in more limited anterior expansion, potentially offering greater stability (13, 18).

The significant difference observed in arch depth (AD) (*p* = 0.036), may reflect a redistribution of teeth along the arch and palatal movement of the central incisors, as reported by Levrini et al. (3).

The analysis of the Intermolar Angle (IMA) revealed no statistically significant differences between the Hyrax and IPE groups at T0 (*p* = 0.872) and at T1 (*p* = 0.433), or in *Δ*IMA (*p* = 0.070). These outcomes align with existing evidence showing that molar buccal tipping is a consistent dentoalveolar response to transverse expansion, as previously described by Bratu (39), Alves et al. (36), and Bruni (19) (18, 34, 36).

Furthermore, no significant differences were observed between the Hyrax and IPE groups regarding changes in palatal depth (PD). In the sample, PD decreased slightly by −0.82 mm in the Hyrax group and by −0.66 mm in the IPE group, values in line with what was reported by Alves (36); Bruni (19), however, observed a modest reduction in PD after RME. In both cases, attributed to the downward displacement of the palatal process following the outward tipping of the maxillary halves (18, 34).

Overall, the available data suggest that the Hyrax remains an effective device for achieving significant transverse skeletal expansion. However, the removable IPE could represent a valid alternative in mixed dentition patients, with the advantage of requiring less parental intervention and having a lower impact on periodontal health. The possibility to remove the device facilitates adequate oral hygiene, reducing plaque accumulation and gingival inflammation, known risk factors for gingival recessions and attachment loss during fixed orthodontic treatment (39–41). Previous studies have shown that removable appliances and clear aligners are associated with better periodontal health compared to fixed appliances, particularly in subjects at higher risk of gingivitis (41, 42).

To assess patient comfort, a questionnaire was administered evaluating several parameters (e.g., dysphonia, dysphagia). Scores were slightly higher in the Hyrax group compared to the IPE group, indicating marginally greater discomfort. These findings align with Rabah (4), who reported greater initial discomfort in patients treated with a rapid expander compared to those treated with a removable slow expander (4).

Nevertheless, certain limitations of the present study should be acknowledged. First, being a pilot study, the results must be interpreted with caution, this type of design may lead a possible overestimation of the observed effects and non-objective interpretation of the results. Also, the relatively small sample size limits the statistical power of the analysis and reduces the the generalizable nature of the findings, particularly with respect to interindividual variability in skeletal and dentoalveolar responses to maxillary expansion. In addition, the short follow-up period, restricted to the immediate post-expansion phase, does not allow conclusions regarding the long-term stability of the observed effects. Second, although occlusal radiography represents a validated, minimally invasive, and clinically reproducible method for assessing midpalatal suture opening, the absence of three-dimensional imaging precludes more detailed evaluation of transverse skeletal changes and their spatial distribution. Moreover, pain was not assessed among the patient-reported outcomes. While the questionnaire focused on functional and mechanical aspects related to the appliances, the inclusion of pain assessment could have provided complementary information on patient experience. Future studies with larger cohorts, long-term follow-up, three-dimensional imaging, and a more comprehensive evaluation of patient-reported outcomes and biomechanical force distribution are needed to further clarify the effects, stability, and mechanisms of action of digital and conventional palatal expanders.

## Conclusions

The preliminary results of this pilot study shows that IPE appears effective in increasing the width of the maxillary arch and promoting the opening of the midpalatal suture. The observed outcomes were largely comparable to those achieved with rapid maxillary expansion using a Hyrax expander for most occlusal parameters and for all radiographic parameters evaluated. While the magnitude of expansion obtained with IPE was slightly lower, the expansion pattern appeared more uniform, consistent with a gradual and controlled activation protocol. Based on the preliminary results and considering the limitations of this pilot study, IPE may be considered a potential alternative to conventional expanders in patients with mixed dentition. Further studies are needed to confirm these results and fully evaluate the clinical applicability, considering larger samples and longer follow-up times.

## Data Availability

The original contributions presented in the study are included in the article/Supplementary Material, further inquiries can be directed to the corresponding author.
