# Supplementary material for: Skeletal and dentoalveolar effects on the midpalatal suture and maxillary arch assessed by occlusal radiographs and three-dimensional digital models in patients treated with invisalign palatal expander and rapid palatal expander: a pilot study
Source: Front Dent Med. 2026 Mar 18;7:1757094. doi: 10.3389/fdmed.2026.1757094 (PMC13038861; doi:10.3389/fdmed.2026.1757094)
Supplement: Supplementary file 1 [file Datasheet1.docx]

SUPPLEMENTARY TABLE S1-Radiographic parameters in the Hyrax group

| Hyrax Group | | | | |
| --- | --- | --- | --- | --- |
| Parameter | Mean ± DS | Median | Min – Max | CI 95% |
| T0 suture | 0.00 ± 0.00 | 0.00 | 0.00 – 0.00 | 0.00 – 0.00 |
| T0 margin | 1.89 ± 0.25 | 1.90 | 1.40 – 2.20 | 1.75 – 2.02 |
| T1 suture | 0.59 ± 0.28 | 0.70 | 0.10 – 1.00 | 0.43 – 0.74 |
| T1 margin | 1.84 ± 0.41 | 1.90 | 1.00 – 2.50 | 1.62 – 2.06 |
| Real margin | 8.28 ± 0.89 | 8.38 | 5.99 – 9.25 | 7.79 – 8.78 |
| T0 real suture | 0.00 ± 0.00 | 0.00 | 0.00 – 0.00 | 0.00 – 0.00 |
| T1 real suture | 2.67 ± 1.20 | 2.78 | 0.37 – 4.28 | 2.00 – 3.33 |
| Expansion | 6.23 ± 1.54 | 6.25 | 4.00 – 8.75 | 5.38 – 7.08 |
| Expansion ratio | 0.46 ± 0.26 | 0.44 | 0.05 – 1.06 | 0.32 – 0.60 |
| Δrx / screw turns number | 0.02 ± 0.01 | 0.03 | 0.00 – 0.05 | 0.02 – 0.03 |
| Δsuture / screw turns number | 0.12 ± 0.06 | 0.11 | 0.01 – 0.27 | 0.08 – 0.15 |

SUPPLEMENTARY TABLE S2-Radiographic parameters in the IPE group

| IPE Group | | | | |
| --- | --- | --- | --- | --- |
| Parameter | Mean ± DS | Median | Min – Max | CI 95% |
| T0 suture | 0.00 ± 0.00 | 0.00 | 0.00 – 0.00 | 0.00 – 0.00 |
| T0 margin | 1.81 ± 0.26 | 1.70 | 1.60 – 2.70 | 1.67 – 1.96 |
| T1 suture | 0.47 ± 0.18 | 0.40 | 0.10 – 0.80 | 0.36 – 0.57 |
| T1 margin | 1.76 ± 0.22 | 1.70 | 1.50 – 2.40 | 1.64 – 1.88 |
| Real margin | 8.04 ± 0.61 | 7.98 | 7.14 – 9.55 | 7.70 – 8.38 |
| T0 real suture | 0.00 ± 0.00 | 0.00 | 0.00 – 0.00 | 0.00 – 0.00 |
| T1 real suture | 2.12 ± 0.79 | 2.02 | 0.46 – 3.34 | 1.69 – 2.56 |
| Expansion | 5.32 ± 0.97 | 5.00 | 4.25 – 7.50 | 4.78 – 5.85 |
| Expansion ratio | 0.40 ± 0.13 | 0.38 | 0.10 – 0.58 | 0.32 – 0.47 |
| Δrx/ ipe devices | 0.02 ± 0.01 | 0.02 | 0.01 – 0.03 | 0.02 – 0.03 |
| Δsuture/ipe devices | 0.10 ± 0.03 | 0.10 | 0.03 – 0.15 | 0.08 – 0.12 |

SUPPLEMENTARY TABLE S3-Occlusal Parameters in Hyrax group

| Hyrax Group | | | | | | |
| --- | --- | --- | --- | --- | --- | --- |
| Parameter | Mean± SD | Median | Min–Max | CI 95% | Shapiro–Wilk W | p(Shapiro–Wilk) |
| CGW | 23.86 ± 1.92 | 23.73 | 21.00 – 26.36 | 22.79 – 24.92 | 0.90 | 0.11 |
| FDMGW | 25.37 ± 2.04 | 25.59 | 21.64 – 28.04 | 24.24 – 26.50 | 0.93 | 0.31 |
| SDMGW | 28.25 ± 2.08 | 28.59 | 24.20 – 32.15 | 27.10 – 29.40 | 0.96 | 0.65 |
| FPMGW | 31.02 ± 1.40 | 30.84 | 28.76 – 33.61 | 30.25 – 31.80 | 0.97 | 0.90 |
| CDW | 31.02 ± 2.07 | 30.58 | 27.22 – 35.33 | 29.87 – 32.17 | 0.98 | 0.93 |
| FDMDW | 35.99 ± 1.94 | 36.23 | 32.51 – 39.01 | 34.92 – 37.07 | 0.97 | 0.79 |
| SDMDW | 41.05 ± 2.69 | 41.42 | 35.19 – 45.33 | 39.56 – 42.54 | 0.96 | 0.71 |
| FPMDW | 47.23 ± 2.44 | 46.80 | 43.47 – 50.78 | 45.87 – 48.58 | 0.94 | 0.35 |
| AP | 74.62 ± 5.00 | 74.56 | 67.73 – 86.19 | 71.85 – 77.39 | 0.94 | 0.37 |
| AD | 25.42 ± 3.28 | 24.28 | 20.99 – 31.90 | 23.60 – 27.24 | 0.92 | 0.21 |

SUPPLEMENTARY TABLE S4-Occlusal Parameters in IPE group T0

| IPE Group T0 | | | | | | |
| --- | --- | --- | --- | --- | --- | --- |
| Parameter | Mean± SD | Median | Min–Max | CI 95% | Shapiro–Wilk W | p(Shapiro–Wilk) |
| CGW | 23.77 ± 1.47 | 23.84 | 21.33 – 26.42 | 22.95 – 24.58 | 0.98 | 0.94 |
| FDMGW | 26.05 ± 2.02 | 26.19 | 21.15 – 29.41 | 24.93 – 27.17 | 0.96 | 0.60 |
| SDMGW | 28.69 ± 2.49 | 29.20 | 22.49 – 33.07 | 27.31 – 30.07 | 0.92 | 0.19 |
| FPMGW | 31.49 ± 2.92 | 31.52 | 25.45 – 36.99 | 29.87 – 33.11 | 0.99 | 1.00 |
| CDW | 31.10 ± 2.17 | 31.58 | 26.66 – 33.99 | 29.90 – 32.30 | 0.93 | 0.30 |
| FDMDW | 37.31 ± 2.60 | 37.41 | 31.75 – 41.06 | 35.87 – 38.76 | 0.96 | 0.60 |
| SDMDW | 41.53 ± 3.03 | 41.53 | 34.75 – 46.30 | 39.86 – 43.21 | 0.97 | 0.89 |
| FPMDW | 48.42 ± 3.42 | 47.17 | 42.32 – 55.19 | 46.53 – 50.32 | 0.96 | 0.76 |
| AP | 75.72 ± 2.74 | 75.26 | 71.57 – 81.69 | 74.20 – 77.24 | 0.97 | 0.78 |
| AD | 25.96 ± 2.59 | 26.20 | 21.56 – 30.25 | 24.53 – 27.40 | 0.97 | 0.86 |

SUPPLEMENTARY TABLE S5 -Questionnaire variables in the Hyrax group

| Hyrax Group | | | | | |
| --- | --- | --- | --- | --- | --- |
| Parameter | N | Min | Max | Mean | SD |
| Bulkiness | 15 | 1.00 | 4.00 | 2.26 | 1.09 |
| Tongue impression | 15 | 1.00 | 3.00 | 1.4 | 0.83 |
| Dysphonia | 15 | 1.00 | 4.00 | 2.33 | 0.89 |
| Dysphagia | 15 | 1.00 | 5.00 | 2.73 | 1.03 |
| Gag reflex | 15 | 1.00 | 2.00 | 1.06 | 0.26 |

SUPPLEMENTARY TABLE S6 -Questionnaire variables in the IPE group

| IPE Group | | | | | |
| --- | --- | --- | --- | --- | --- |
| Parameter | N | Min | Max | Mean | SD |
| Bulkiness | 15 | 1.00 | 3.00 | 1.93 | 0.73 |
| Tongue impression | 15 | 1.00 | 1.00 | 1.00 | 0.00 |
| Dysphonia | 15 | 1.00 | 3.00 | 1.93 | 0.92 |
| Dysphagia | 15 | 1.00 | 4.00 | 2.50 | 0.94 |
| Gag reflex | 15 | 1.00 | 3.00 | 1.36 | 0.633 |
